# Multi-omics analysis reveals CLIC1 as a therapeutic vulnerability of gliomas

**DOI:** 10.3389/fphar.2023.1279370

**Published:** 2023-11-08

**Authors:** Chengcheng Wang, Zheng He

**Affiliations:** ^1^ Department of Pharmacy, Qilu Hospital, Cheeloo College of Medicine, Shandong University, Qingdao, Shandong, China; ^2^ Department of Neurosurgery, Qilu Hospital, Cheeloo College of Medicine, Shandong University, Qingdao, Shandong, China

**Keywords:** low-grade gliomas, tumor microenvironment, CLIC1, anticancer immunity, immune-checkpoint blockade, therapeutic vulnerability

## Abstract

**Introduction:** Despite advances in comprehending cancer biology, malignant gliomas remain incurable. The present work conducted a multi-omics analysis for investigating the significance of chloride intracellular channel 1 (CLIC1) in gliomas.

**Methods:** Multi-omics data of glioma covering transcriptomics, genomics, DNA methylation and single-cell transcriptomics from multiple public cohorts were enrolled for analyzing CLIC1. *In vitro* experiments were conducted to measure apoptosis and cell mobility in U251 and U373 glioma cells following transfection of CLIC1 siRNAs.

**Results:** Elevated CLIC1 expression was proven to stably and independently estimate worse survival outcomes. CLIC1 expression was higher in more advanced stage, wild-type IDH and unmethylated MGMT samples. Tumorigenic and anticancer immunity pathways were remarkably enriched in CLIC1-up-regulated tumors. Additionally, CLIC1 was positively linked with cancer-immunity cycle, stromal activation, DNA damage repair and cell cycle. Suppressing CLIC1 resulted in apoptosis and attenuated cell motility of glioma cells. More frequent genomic alterations were found in CLIC1-up-regulated tumors. CLIC1 expression presented a remarkably negative connection to DNA methylation. High CLIC1 expression samples were more sensitive to camptothecin, cisplatin, doxorubicin, erlotinib, paclitaxel, rapamycin, clofarabine, tanespimycin, methotrexate, everolimus, TAK-733, trametinib and AZD8330. Tumors with upregulated CLIC1 presented abundant immune cell infiltration, higher expression of immune-checkpoints and -modulators and similar transcriptome profiling, indicative of well response to immune-checkpoint blockade (ICB). Nevertheless, due to elevated TIDE score, tumors with CLIC1 upregulation appeared to be resistant to ICB. Single-cell analysis unveiled that CLIC1 was expressed ubiquitously in tumor cells and tumor microenvironment.

**Conclusions:** Overall, CLIC1 was a promising treatment vulnerability in glioma.

## Introduction

Gliomas account for nearly 80% of primary malignant brain tumors within the central nervous system (Yang et al., 2022). The most recent 2021 update by the World Health Organization (WHO) has integrated histological, molecular, and genomic characteristics into the systematic classification of gliomas (Louis et al., 2021). As per this classification, gliomas are stratified into four grades, with Grades 1 and 2 categorized as low-grade gliomas (LGG) and Grades 3 and 4 designated as high-grade gliomas (HGG) (Baumert et al., 2016; Buckner et al., 2016; Louis et al., 2021). LGG (Grade 1/2) constitute around 6% of primary brain tumors in the adult population, characterized by a relatively favorable overall survival (OS) of approximately 7 years (McClellan et al., 2023). It is noteworthy, however, that Grade 2 LGG frequently demonstrate a propensity for recurrence and progression to HGG (Stupp et al., 2017). Among HGG, glioblastoma multiforme (GBM) stands out as the most common and clinically aggressive Grade 4 glioma, with a median survival of only 12–15 months, despite standard-of-care therapy (Yu et al., 2022). Hence, the exploration of potential therapeutic vulnerabilities targeting gliomas assumes critical importance.

Chloride channels are a diverse group of proteins, which modulate basic cellular processes, e.g., stabilization of cellular membrane potential, transepithelial transport, maintenance of intracellular pH and cellular volume (Sarkar, 2022; Pressey et al., 2023). Chloride channels have been demonstrated to be responsible for glioma progression (Ozaki et al., 2021). Chloride intracellular channel 1 (CLIC1) is a member of the p64 family. The protein is primarily localized in the nucleus and displays chloride channel activity both in the nuclear and plasma membrane (Hossain et al., 2023). The role of CLIC1 in glioma has been proposed. Increased expression of CLIC1 correlates to unfavorable prognostic outcomes of gliomas (Wang et al., 2012). While CLIC1 activity itself may not be a determinant in the development of GBM (Barbieri et al., 2022), its suppression appears to influence the characteristics of glioma stem cells and their response to novel biguanide derivatives (Peretti et al., 2018). Nevertheless, the formation and activation mechanisms of functional CLIC1 in glioma remains indistinct.

To address these gaps in knowledge, the present study has conducted a comprehensive multi-omics analysis. Our aim is to determine the therapeutic potential of targeting CLIC1 and to unveil the molecular mechanisms underlying its role in gliomas. This research seeks to contribute to a better understanding of the intricate relationship between chloride channels, specifically CLIC1, and glioma progression, which may open doors to new treatment strategies.

## Materials and methods

### Multi-omics data acquisition

RNA sequencing data of LGG and GBM tumors from the Cancer Genome Atlas (TCGA) were retrieved via the Genomic Data Commons (https://portal.gdc.cancer.gov/) by use of TCGAbiolinks package (Colaprico et al., 2016). LGG (*n* = 530) and GBM (*n* = 168) samples were combined and batch effects were corrected utilizing sva package (Leek et al., 2012). Four glioma cohorts: CGGA_325 (*n* = 313) and CGGA_693 (*n* = 657) from the Chinese Glioma Genome Atlas (CGGA; http://www.cgga.org.cn/) (Zhao et al., 2021) and GSE16011 (*n* = 261) (Gravendeel et al., 2009) and GSE43378 (*n* = 50) (Kawaguchi et al., 2013) from the Gene Expression Omnibus (https://www.ncbi.nlm.nih.gov/geo/) were utilized as external verification. Supplementary Table S1 summarizes the clinical traits of above cohorts. GSE138794 dataset from GEO was used for the Single-cell RNA sequencing (ScRNA-seq) analysis. Copy number variations, somatic mutation and DNA methylation data were also curated from the UCSC Xena database (http://xena.ucsc.edu/).

### Functional enrichment analysis

On the basis of the “c5.go.v7.4.symbols” and “c2.cp.kegg.v7.4.symbols” gene sets from the Molecular Signatures Database (Liberzon et al., 2015), gene set enrichment analysis (GSEA) was carried out (Xu et al., 2018). Enrichment values of specific well-established pathways (Tian et al., 2023) were quantified utilizing GSVA package (Xu et al., 2018).

### Cell culture and transfection

Human glioma cell lines U251 and U373 (Cell Bank of the Chinese Academy of Sciences, Shanghai, China) were grown in RPMI-1640 medium (SEVEN, California, United States) with 10% fetal bovine serum (Hyclone, Utah, United States) in a humidified incubator with 5% CO_2_ at 37°C. SiRNAs of CLIC1 (si-CLIC1) and negative control (si-NC) were synthesized by GenePharma (Shanghai, China), which were transfected into cells based upon X-tremeGENE™ siRNA transfection reagent (Roche, New Jersey, United States).

### Real-time quantitative PCR (RT-qPCR)

Total RNA was extracted via Trizol (Yeasen, Shanghai, China). RNA quality was evaluated in line with OD260/OD280 ratio. The extracted RNA was reversely transcribed into cDNA. RT-qPCR was conducted based upon the ABI QuantStudio™ 12K Flex (ABI, Connecticut, United States). Primer sequences included: CLIC1, 5’-AGT​CCC​AGC​AAC​CCA​GAA​TTT-3’ (forward), 5’-CAC​GAA​CAA​TTC​GAC​CTG​CG-3’ (reverse); GAPDH, 5’-GAA​TGG​GCA​GCC​GTT​AGG​AA-3’ (forward), 5’-AAA​AGC​ATC​ACC​CGG​AGG​AG-3’ (reverse). Relative expression of CLIC1 was computed with 2^−ΔΔCT^.

### Western blotting

Whole-cell protein was extracted from U251 and U373 in RIPA buffer (Thermo Fisher Scientific, United States) and centrifuged at 12,000 rpm for 20 min. A BCA kit (Thermo, Waltham, MA, 23228) was used to measure the protein concentration. After immunoblotting, the proteins were transferred to a nitrocellulose membrane and incubated with specific antibodies. The following primary antibodies were used: β-actin (Proteintech,60008-1-lg), CLIC1 (Cell Signaling Technology, D7D6H Rabbit mAb #53424).

### Flow cytometry

Cell apoptosis was measured via cell apoptosis detection kit (Abbkine, Wuhan, China). The supernatant was absorbed from the culture plate and transfered it to the centrifuge tube for preservation. The cells were washed twice with PBS, and the cleaning solution was collected for preservation. Appropriate amount of PBS was used to blow down the cells, the cell suspension was transferred to the centrifuge tube, and the supernatant of step 1 and cleaning solution of step 2 were added together. Following centrifugation at 1,000 rpm for 5 min and discard the supernatant. After adding PBS, centrifuge at 1,000 rpm for 5 min, the supernatant was discarded. This step was repeated. A cell suspension of 1 × 10*6 cells/ml was prepared via adding the pre-prepared 1× Annexin V buffer. 100 μl of cell suspension was taken and added to the new tube. The cell suspension was added with 5 μl Annexin V-AbFluor ™ 488 binding and 2 μl PI, and incubated at room temperature away from light for 15 min. 400 μl 1×Annexin V buffer was added, and apoptosis was tested within 1 h.

### Wound healing assay

Cell motility was detected via wound healing assay. Cells were seeded in 6-well plates. When the cell confluence reached 100%, wounds were produced utilizing a 200-μL micropipette tip. The scratched cells were discarded and serum-free medium was added. Photographs were acquired at 0 and 48 h by use of an inverted microscope (Olympus, Japan).

### Genomic alteration evaluation

Copy number gains and losses were estimated by use of GISTIC 2.0 (Mermel et al., 2011). Somatically mutated genes were assessed via maftools computational approach (Mayakonda et al., 2018). Tumor mutational burden (TMB), aneuploidy score, cancer testis antigen (CTA), fraction of genome altered and number of segments of glioma specimens were also analyzed.

### Drug sensitivity estimation

By pRRophetic package (Geeleher et al., 2014), half-maximal inhibitory concentration (IC50) values of commonly applied drugs were estimated in accordance with the Genomics of Drug Sensitivity in Cancer cell line expression spectrum (Yang et al., 2013). After gathering drug sensitivity data from the CTRP and PRISM datasets, response to small molecular compounds was evaluated based upon area under the curve (AUC) (Ghandi et al., 2019).

### Anticancer immunity assessment

Activity of steps in the cancer-immunity cycle (Chen and Mellman) was estimated by use of GSVA package (Xu et al., 2018). Expression of chemokine, receptor, immuno-stimulator, MHC and immune checkpoint molecules was computed (Chen et al., 2021). Through single-sample GSEA (ssGSEA) (Hanzelmann et al., 2013), infiltration of immune cell types was estimated. Response to PD-1 or CTLA4 antibody (Chen et al., 2016; Balar et al., 2017) was inferred based upon subclass mapping (Submap) algorithm (Hoshida et al., 2007). Tumor Immune Dysfunction and Exclusion (TIDE) (Jiang et al., 2018) was employed for estimation of immune-checkpoint blockade (ICB) efficacy.

### ScRNA-seq analysis

ScRNA-seq data from nine glioma specimens were acquired from the GSE138794 dataset (Wang et al., 2019). Quality control was implemented, with subsequent removal of cells with >10% mitochondrial UMI counts. The analysis was achieved through Seurat package (Butler et al., 2018). The top 2,000 genes with high variability were chosen. Cell types were then clustered and recognized based upon known cell markers that were gathered from the CellMarker database (Zhang et al., 2019). Function role of CLIC1 in the single cell level was investigate through GSVA R package (Hanzelmann et al., 2013). The CellChat R package (v1.6.1) was used for cell-cell communication analysis. Glioma cells were isolated from all cells and categorized into “astrocyte,” “oligodendrocyte,” “macrophage,” “glial cell,” “monocyte,” and “endothelial cell.” A CellChat object was then created with the CellChat function (Jin et al., 2021). The computeCommunProbPathway function yielded cell-to-cell interactions for each cell signaling pathway. The CytoTRACE algorithm assessed cell differentiation and developmental potential by analyzing factors like mRNA feature expression and distribution (Gulati et al., 2020). To identify the initial stage of cellular differentiation, we used the CytoTRACE R package (v0.3.3). Additionally, we employed pseudotime analysis from the monocle2 R package (v2.24.0) (26) to determine the direction of cellular differentiation (Qiu et al., 2017).

### Statistical analysis

**FIGURE 1:**
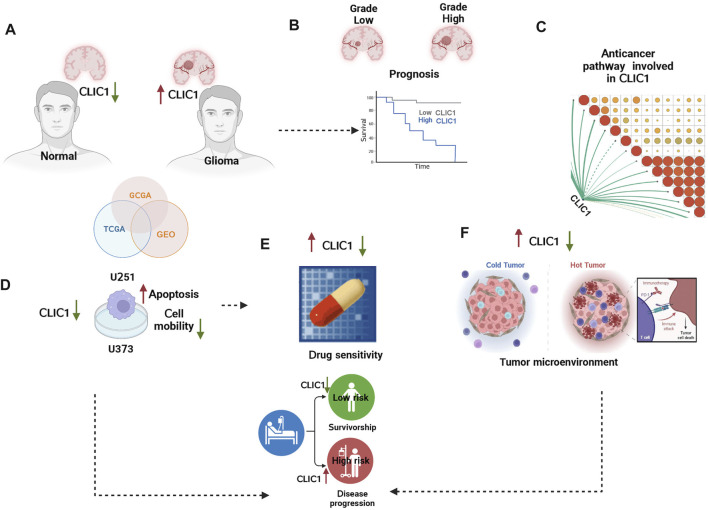
The graphical abstract and analysis flow chart of present study. **(A)** Multi-omics data acquisition. Gliomas present high expression of CLIC1. **(B)** Prognostic implication of CLIC1 was investigated in gliomas. **(C)** Signaling pathways involved in CLIC1. **(D)** Experiments validated CLIC1 as a therapeutic vulnerability of gliomas. **(E)** Association of CLIC1 with drug sensitivity. **(F)** Anticancer immunity assessment of CLIC1.

All the analyses were achieved via appropriate R packages (version 4.2.1). OS, disease-specific survival (DSS) and progression-free survival (PFS) analyses were conducted through Kaplan–Meier curves and log-rank test utilizing survival package. Receiver operating characteristic curves (ROCs) were drawn for appraising the prediction efficiency via pROC package, and AUC values were computed. Continuous data between two groups were compared utilizing student’s t or Wilcoxon rank-sum test, with one-way analysis of variance for comparing ≥3 groups. Through uni- and multivariate cox regression methods, association of variables with survival was investigated. Pearson’s test was adopted for correlation analysis. Statistical significance was set as *p* < 0.05. Figure 1 presents the whole analysis flow chart.

## Results

### Upregulated CLIC1 correlates to undesirable prognostic outcomes of glioma

**FIGURE 2:**
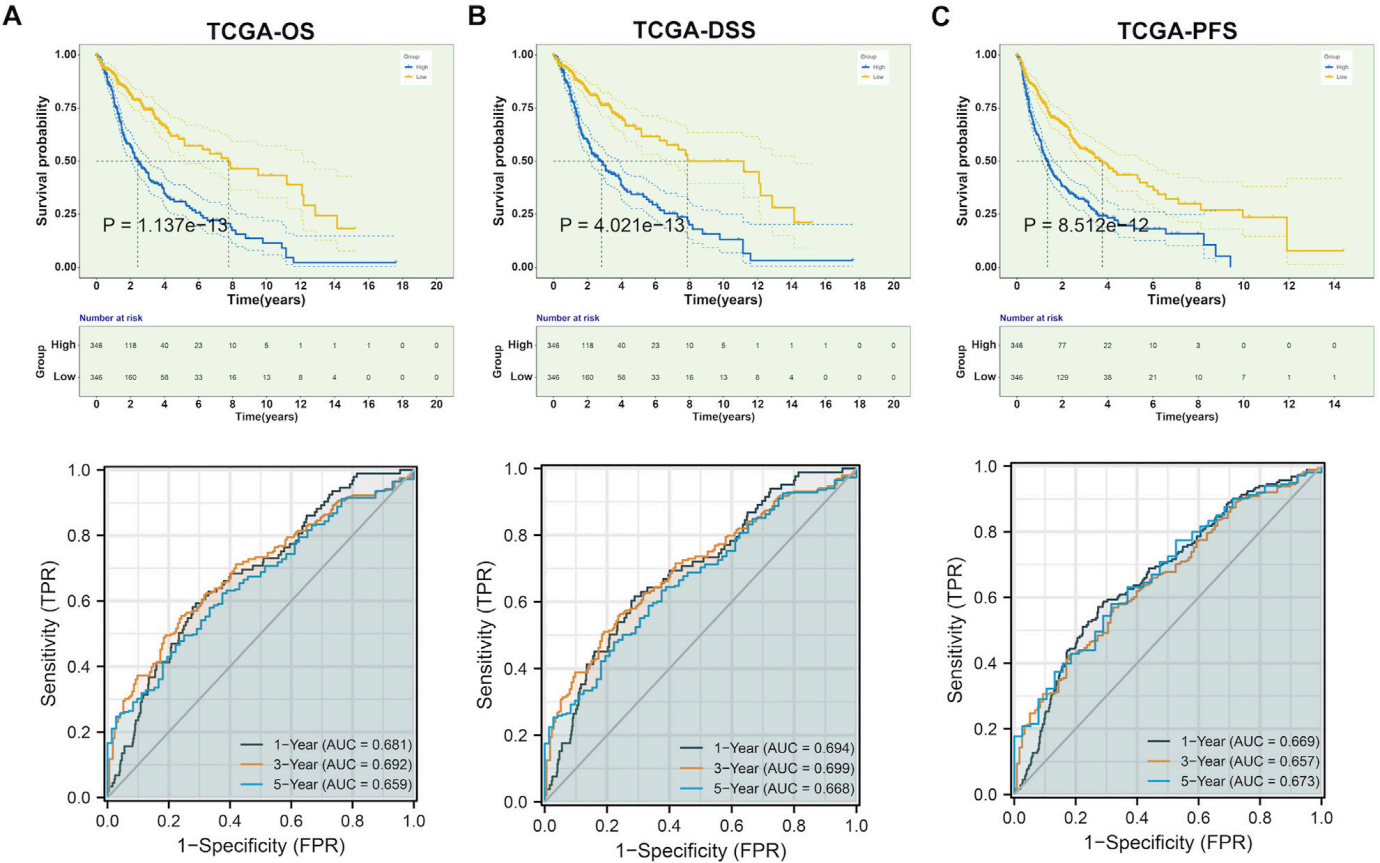
Upregulated CLIC1 correlates to undesirable prognostic outcomes of glioma. **(A–C)** Different OS, DSS and PFS outcomes in lowly and highly expressed CLIC1 patients as well as one-, three- and 5-year ROCs in TCGA cohort. OS, DSS and PFS analyses were conducted through Kaplan–Meier curves and log-rank test utilizing survival package. OS, overall survival; DSS, disease-specific survival; PFS, progression-free survival; ROC, receiver operating characteristic.

The study integrated TCGA-GBM and TCGA-LGG samples and corrected batch effects for analyzing CLIC1 in glioma (Supplementary Figures S1A, B). Prognostic implication of CLIC1 was firstly investigated in glioma. Patients with CLIC1 upregulation presented poorer OS outcomes versus those with CLIC1 downregulation (Figure 2A). ROCs were utilized for verifying the efficacy of CLIC1 in prognostication. AUC values at one-, three- and 5-year OS exceeded 0.65, demonstrating that CLIC1 enabled to potentially estimate OS. Association of CLIC1 with DSS and PFS was also evaluated. Worse DSS and PFS outcomes were found in patients with high CLIC1 expression (Figures 2B, C). AUC values of one-, three- and 5-year DSS and PFS were found to be more than 0.65, indicative of the efficacy of CLIC1 in estimation of DSS and PFS. For verifying the reliability and reproducibility of CLIC1 in prognostication, four cohorts: CGGA_325, CGGA_693, GSE16011 and GSE43378 were adopted. Consistently, upregulated CLIC1 was connected to terrible OS outcomes and presented the satisfactory efficiency of CLIC1 in survival estimation in above cohorts (Supplementary Figures S2A–D).

### CLIC1 is linked with glioma patients’ clinicopathological traits

**FIGURE 3:**
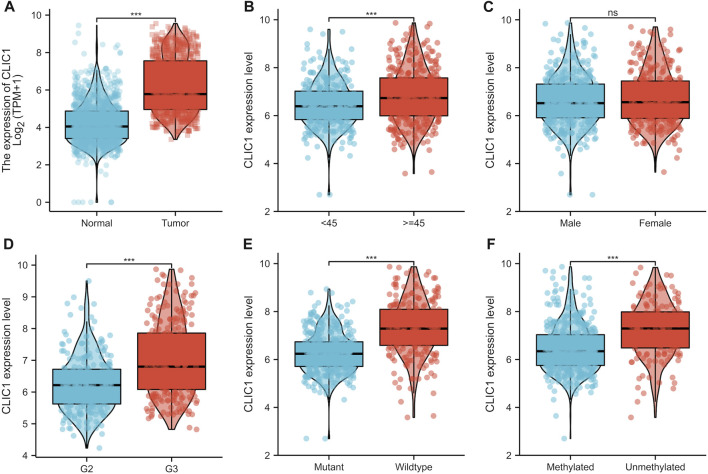
CLIC1 is linked with glioma patients’ clinicopathological traits. **(A)** Comparison of CLIC1 expression in glioma and normal tissues. **(B–F)** Comparison of CLIC1 expression in age <45 versus ≥45; male versus female; grade II (G2) versus grade III (G3); wild-type versus mutant IDH1; methylated versus unmethylated MGMT specimens. The Student’s t-test was used to compare the statistical difference between two groups. ***p-value < 0.001; ns: no significance.

Although CLIC1 is expressed ubiquitously in human tissues (Wang et al., 2012), CLIC1 was found to present aberrant overexpression in glioma versus normal tissues (Figure 3A). Association of CLIC1 with clinicopathological features was subsequently assessed. CLIC1 expression was remarkably higher in age ≥45 than <45 (Figure 3B). No significant difference was detected in male and female specimens (Figure 3C). CLIC1 was also detected to exhibit higher expression in grade III versus II (Figure 3D), indicating that CLIC1 upregulation was connected to more advanced grade. Mutant IDH1 and O^6^-methylguanine DNA methyltransferase (MGMT) promoter methylation associate with well prognostic outcomes of glioma patients (Della Monica et al., 2022). CLIC1 expression was notably higher in wild-type than mutant IDH1 tumors (Figure 3E). Additionally, unmethylated MGMT tumors displayed higher CLIC1 expression versus those with methylated MGMT (Figure 3F).

### CLIC1 independently estimates glioma patients’ prognosis

Integration of uni- and multivariate cox regression analyses showed that CLIC1 was independently predictive of patient survival in TCGA cohort (Supplementary Figures S3A, B). The independency of CLIC1 in prognostication was proven in the CGGA_325 and CGGA_693 datasets (Supplementary Figures S3C–F).

### Signaling pathways and anticancer immunity involved in CLIC1

**FIGURE 4:**
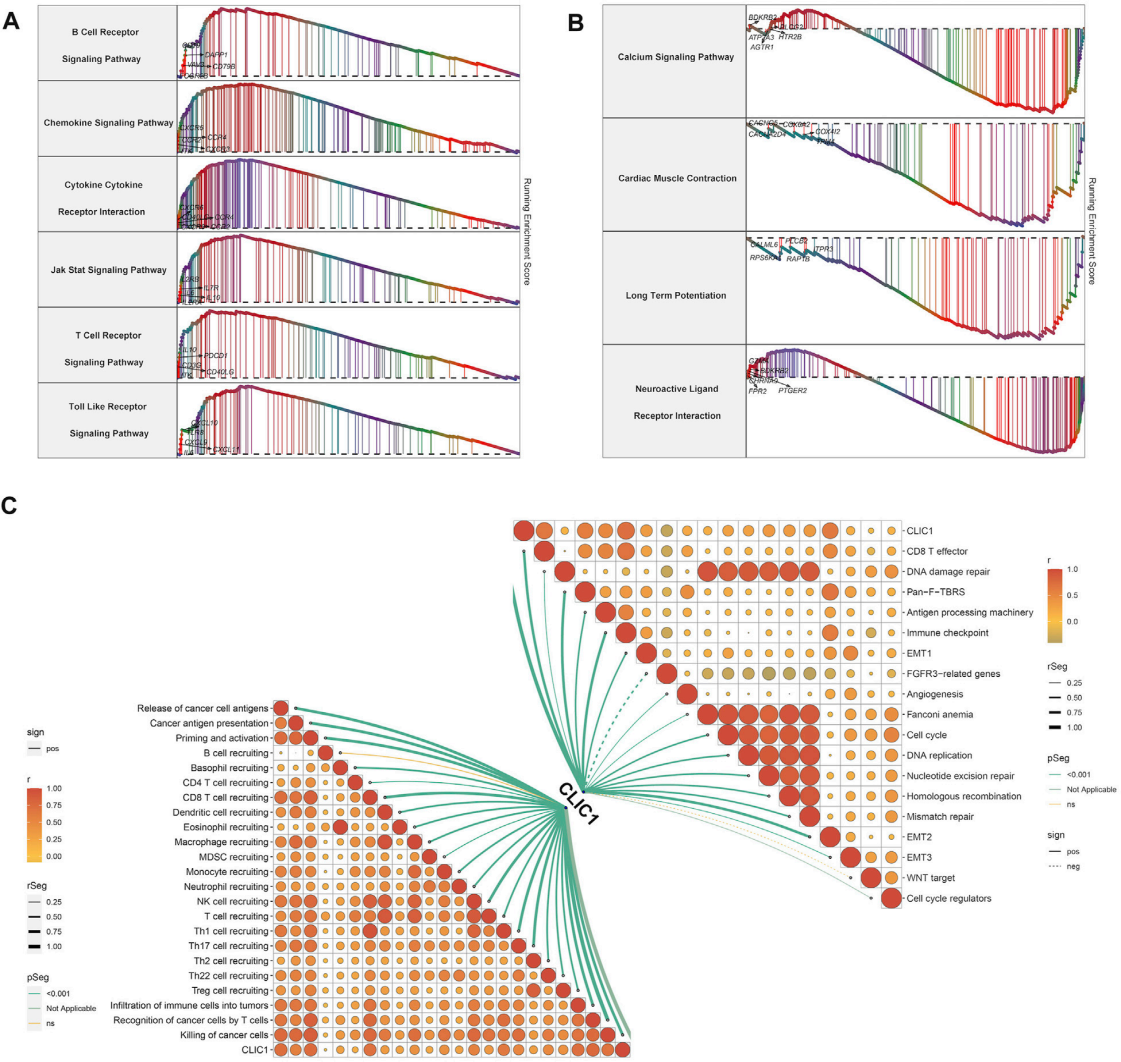
Signaling pathways involved in CLIC1. **(A)** Enrichment of GO and KEGG pathways in high CLIC1 expression tumors. **(B)** Enrichment of GO and KEGG pathways in low CLIC1 expression tumors. **(C)** Correlation of CLIC1 with cancer-immunity cycle and well-established pathways.

Tumorigenic and immunity-relevant signaling pathways: B cell receptor, chemokine, cytokine-cytokine receptor, JAK-STAT, T cell receptor and Toll-like receptor exhibited the remarkable enrichment in upregulated CLIC1 tumors (Figure 4A). Physiological processes, e.g., calcium signaling pathway, cardiac muscle contraction, long-term potentiation and neuroactive ligand receptor interaction were enriched in downregulated CLIC1 tumors (Figure 4B). In addition, CLIC1 was identified to positively associate with anticancer immunity (e.g., CD8 T effector, antigen processing machinery and immune checkpoint), cell cycle, stromal activation (pan-fibroblast TGFβ response signature (Pan-F-TBRS), epithelial-mesenchymal transition (EMT)1∼3 and angiogenesis) and DNA damage repair mechanisms (DNA replication, nucleotide excision repair, homologous recombination and mismatch repair) (Figure 4C). Interrupting one or more steps within the cancer immune cycle allows the tumor to evade immune surveillance (Somarribas Patterson and Vardhana, 2021). CLIC1 presented positive interactions with all steps in the cancer immune cycle (Figure 4C), proving the significance of CLIC1 in modulating anticancer immunity.

### CLIC1 is a therapeutic vulnerability of glioma

**FIGURE 5:**
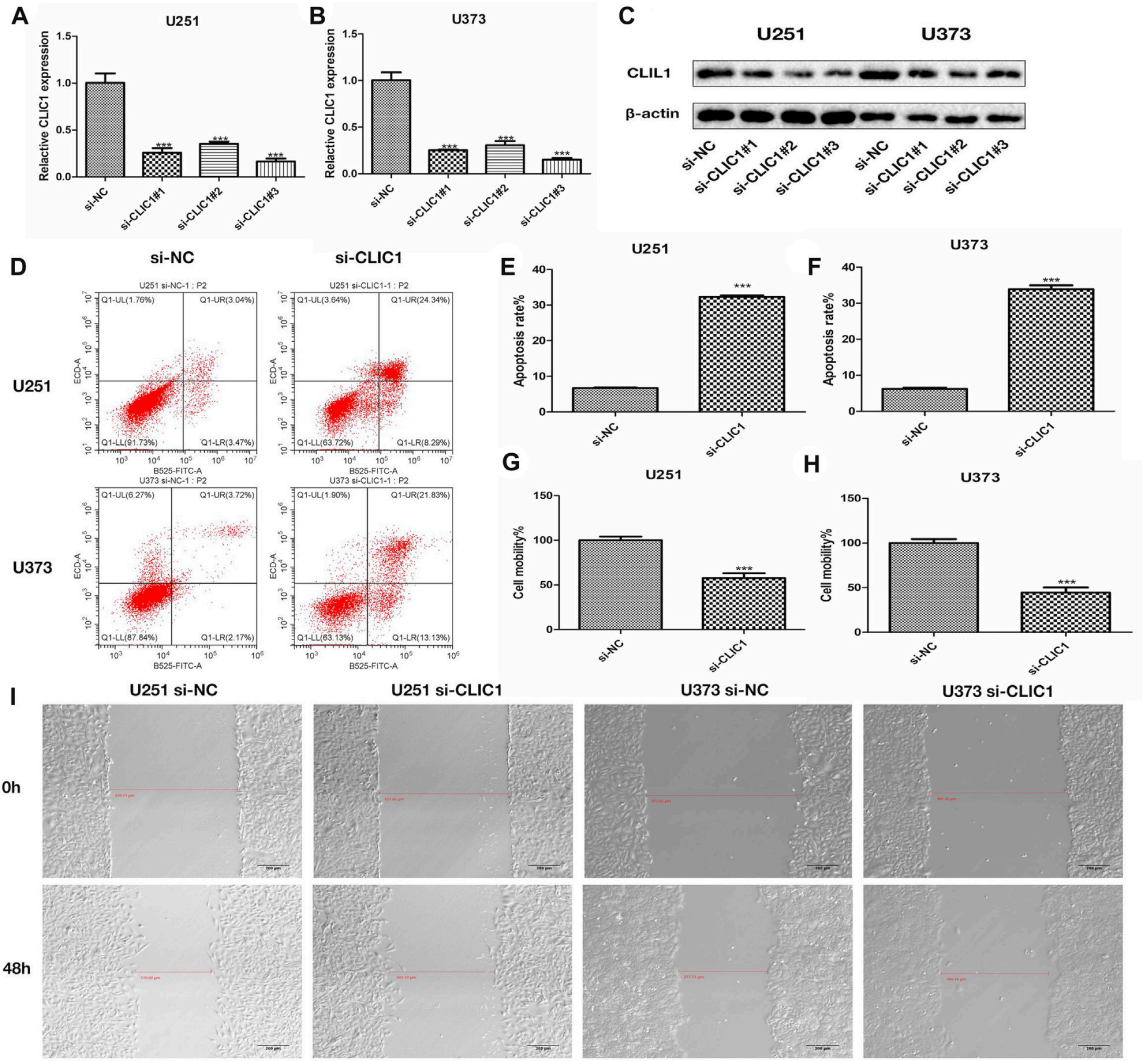
CLIC1 is a therapeutic vulnerability of glioma. **(A,B)** RT-qPCR for measurement of CLIC1 expression in U251 and U373 cells transfected with si-NC or si-CLIC1. **(C)** The protein level of the CLIC1 in glioma cells transfected with si-CLIC1 and a control sequence were assessed by Western blotting. β-actin was the loading control. **(D–F)** Flow cytometry for detection of apoptosis of si-NC or si-CLIC1-transfected U251 and U373 cells. **(G–I)** Wound healing for evaluation of cell mobility of si-NC- or si-CLIC1-transfected U251 and U373 cells. The Student’s t-test and one-way analysis of variance (ANOVA) were respectively used to compare the statistical differences between two groups and three or more groups. Bar, 200 μm. ***p-value < 0.001.

The study then assessed the potential of CLIC1 as a therapeutic vulnerability of glioma. U251 and U373 glioma cells were transfected with si-CLIC1 to silence CLIC1 expression (Figures 5A, B). The protein level of the CLIC1 in glioma cells transfected with si-CLIC1 and a control sequence were assessed by Western blotting (Figure 5C). Based upon flow cytometry results, CLIC1-silent U251 and U373 cells presented remarkable enhancement in apoptosis (Figures 5D–F). In addition, wound healing results demonstrated that CLIC1 knockdown prominently alleviated cell mobility of U251 and U373 cells (Figures 5G–I). Overall, CLIC1 was regarded as a therapeutic vulnerability of glioma.

### Genomic alterations and DNA methylation associated with CLIC1

**FIGURE 6:**
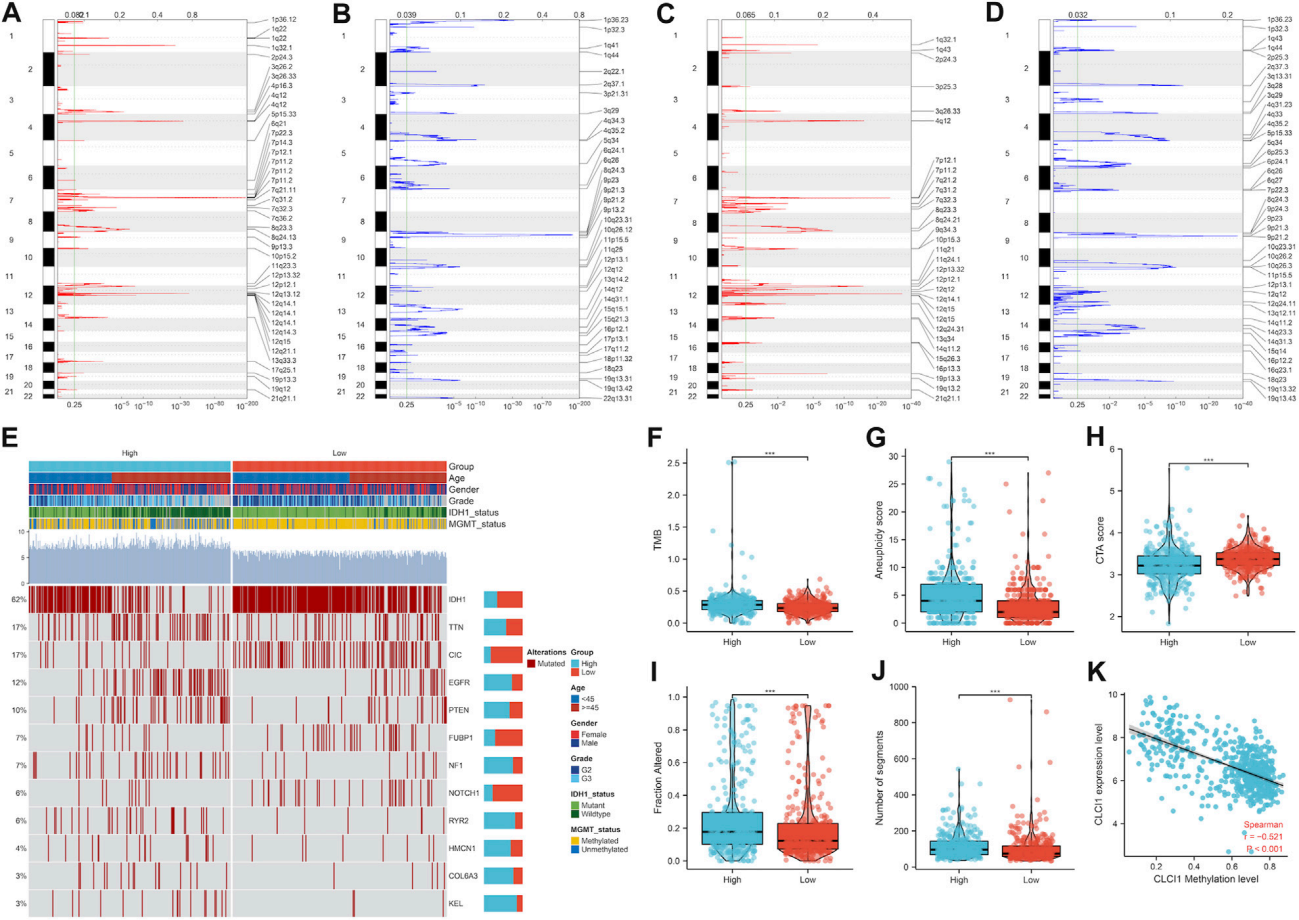
Genomic alterations and DNA methylation associated with CLIC1. **(A,B)** Gene gains and losses in glioma tumors with upregulated CLIC1. Red, gains; blue, losses. The significant cutoff was set as q-value of 0.25. **(C,D)** Gene gains and losses in tumors with downregulated CLIC1. **(E)** Somatically mutant genes in highly or lowly expressed CLIC1 tumors. Genes are ranked by mutant frequency. **(F–J)** Different TMB, aneuploidy score, CTA score, fraction of genome altered and number of segments in two groups. **(K)** Correlation between CLIC1 methylation and its expression across glioma tumors. The Student’s t-test was used to compare the differences between two groups in terms of TMB, aneuploidy score, CTA score, fraction of genome altered, and the number of segments. The correlation between CLIC1 methylation and its expression across glioma tumors was assessed using the Spearman test. ***p-value < 0.001.

More frequent gene gains and losses were detected in CLIC1 upregulation tumors (Figures 6A, B) in comparison to those with CLIC1 downregulation (Figures 6C, D). In addition, heterogeneous somatic mutations were observed in CLIC1 low and high tumors, e.g., IDH1 (78.8% versus 43.4%), CIC (26.6% versus 6%), NOTCH1 (9.3% versus 2.8%) and FUBP1 (9.6% versus 4.1%) occurred more frequent mutations in low CLIC1 tumors, and EGFR (6.6% versus 18.4%), RYR2 (2.1% versus 9.2%), with more frequently mutant NF1 (3.3% versus 10.4%), KEL (0.9% versus 5.4%), PTEN (6.9% versus 14.2%) and COL6A3 (1.5% versus 5.1%) in high CLIC1 tumors (Figure 6E). TMB and aneuploidy score were detected to be notably higher in CLIC1-up-regulated tumors, indicative of more mutations (Figures 6F, G). Additionally, high CLIC1 expression tumors presented lower CTA score (Figure 6H) as well as higher fraction of genome altered and number of segments (Figures 6I, J) versus those with lowly expressed CLIC1. Thus, CLIC1 upregulation was connected to more frequently genomic alterations. It was also found the prominently negative interaction of CLIC1 methylation with its expression (Figure 6K), indicating that CLIC1 overexpression was potentially affected by its hypomethylation.

### Association of CLIC1 with drug sensitivity

**FIGURE 7:**
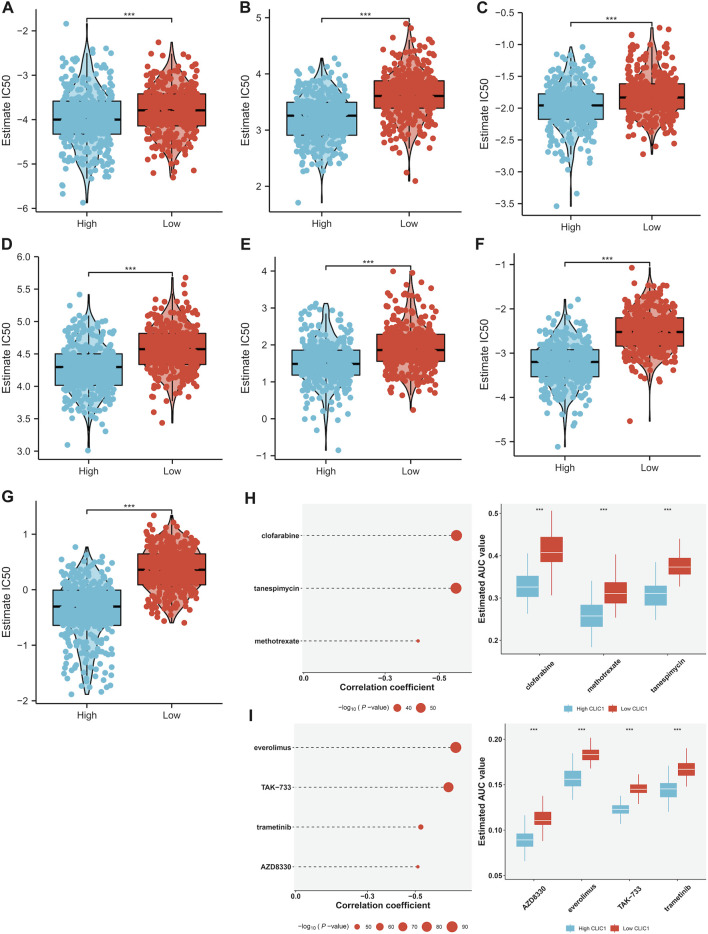
Association of CLIC1 with drug sensitivity. **(A–G)** Different IC50 values of **(A)** camptothecin, **(B)** cisplatin, **(C)** doxorubicin, **(D)** erlotinib, **(E)** etoposide, **(F)** paclitaxel and **(G)** rapamycin in highly and lowly expressed CLIC1 tumors. **(H,I)** Association of CLIC1 with AUC values of **(H)** CTRP- and **(I)** PRISM-derived drugs and different AUC values between down- and upregulated CLIC1 samples. The Student’s t-test was used to compare the statistical difference between two groups. ***p-value < 0.001.

Tumors with high CLIC1 expression displayed prominently lower IC50 values of camptothecin, cisplatin, doxorubicin, erlotinib, paclitaxel and rapamycin in comparison to those with lowly expressed CLIC1 (Figures 7A–G), suggesting that CLIC1 upregulation was linked with sensitivity to these drugs. CLIC1 was found to negatively correlate to AUC of CTRP compounds: clofarabine, tanespimycin and methotrexate; furthermore, highly expressed CLIC1 tumors exhibited lower AUC (Figure 7H). CLIC1 was also negatively connected to AUC of PRISM compounds: everolimus, TAK-733, trametinib and AZD8330, with lower AUC in high CLIC1 expression tumors (Figure 7I). Above compounds might be appropriate for patients with overexpressed CLIC1.

### CLIC1 upregulation is linked with hot tumors

Nearly all immune cell types (notably T cells) showed richer infiltration in CLIC1-up-regulated tumors (Supplementary Figure S4A). In addition, CLIC1 was positively connected to immune cell infiltration (Supplementary Figure S4B). With the increase in CLIC1 expression, expression of chemokine, receptor, immuno-stimulator and MHC molecules was gradually elevated (Supplementary Figures S4C–F). These data unveiled that CLIC1 upregulation was in relation to hot tumors.

### Upregulated CLIC1 associates with response to ICB

**FIGURE 8:**
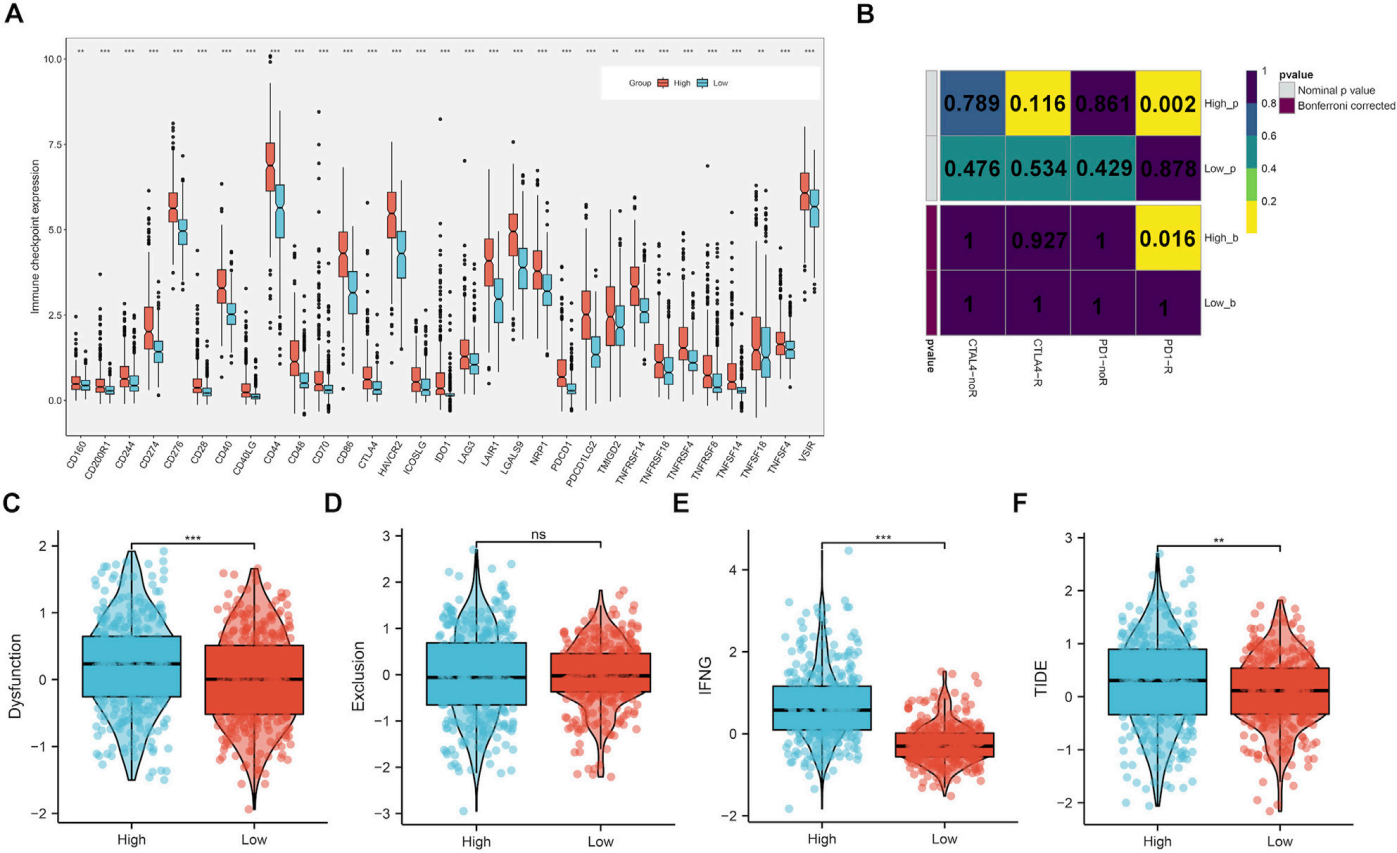
Upregulated CLIC1 associates with response to ICB. **(A)** Expression of immune checkpoints in lowly and highly expressed CLIC1 tumors. **(B)** Submap for evaluating the similarity in expression profiles between low or high CLIC1 expression samples and response to PD-1 or CTLA4 antibody. **(C–F)** Comparison of dysfunction, exclusion, IFNG and TIDE scores in tumors with up- and downregulated CLIC1. The Wilcoxon rank-sum test was used to compare the differences between two groups. **p-value < 0.01; ***p-value < 0.001; ns, not significant.

Samples with highly expressed CLIC1 exhibited remarkably higher levels of almost all immune checkpoints (such as CD40/CD40LG, PDCD1/PDCD1LG2, CTLA4, CD276 and IDO1) versus those with low CLIC1 expression (Figure 8A). In addition, high CLIC1 expression tumors presented the similar expression profiling to patients who responded to PD-1 antibody (Figure 8B). TIDE approach was utilized for estimating the efficacy of ICB. Consequently, dysfunction, IFNG and TIDE scores were prominently higher in CLIC1-up-regulated tumors, without difference in exclusion score (Figures 8C–F), indicative of resistance to ICB. Hence, although tumors with CLIC1 upregulation had highly expressed immune checkpoints and abundant immune cells, they were resistant to ICB.

### Single-cell analysis of CLIC1 in glioma tumors

**FIGURE 9:**
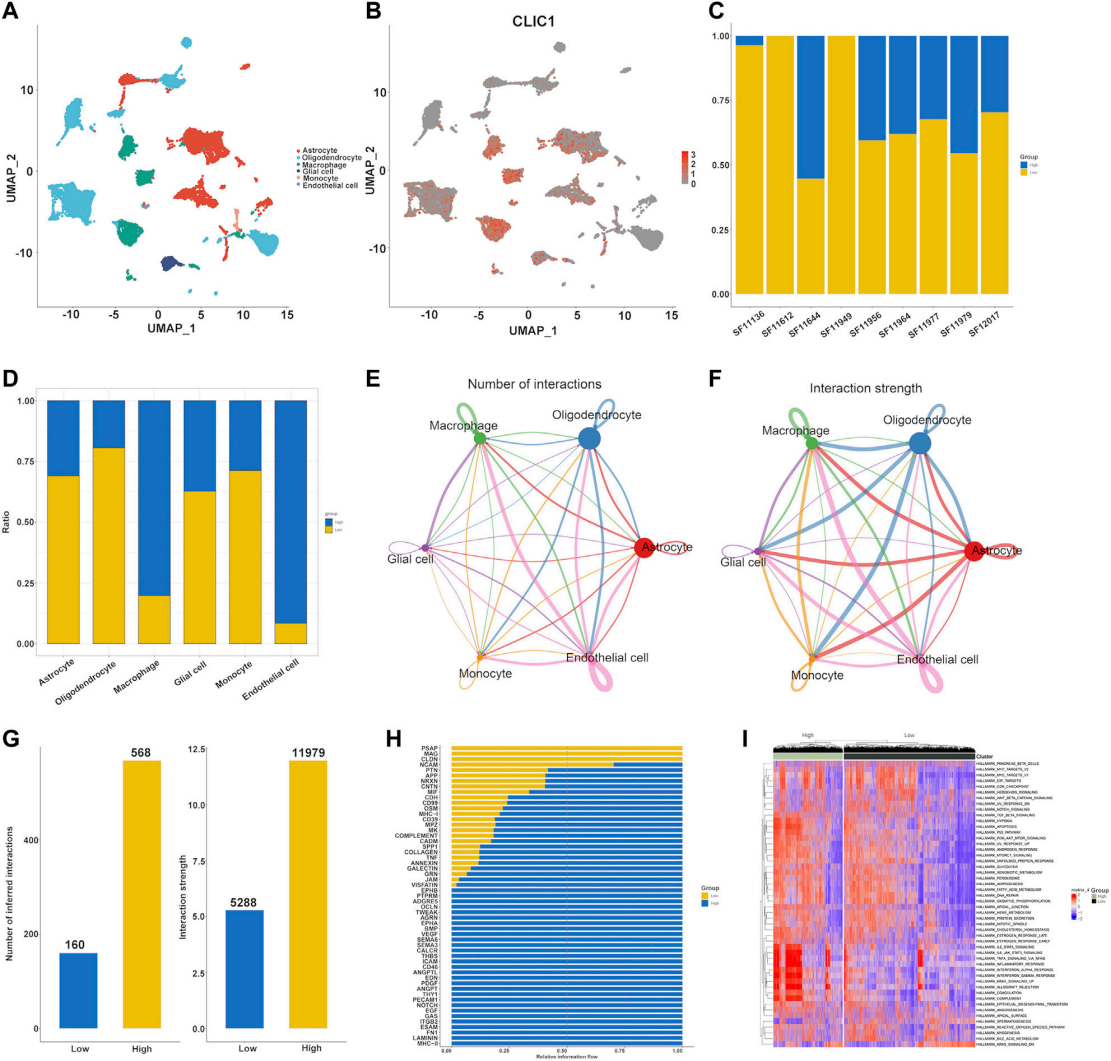
Single cell analysis of CLIC1 in Golima tumors. **(A)** Identification of cell types in line with well-established markers. **(B)** Expression distribution of CLIC1 in distinct cell types. **(C)** Proportion of high and low CLIC1 group in golima patients. **(D)** Proportion of high and low CLIC1 group in cell types. The landscape of golima’s intercellular communication between each cell type is represented by the thickness of the line, which symbolizes the number of ligand-receptor pairs **(E)** or interaction weight **(F)**. **(G)** Comparisons of inferred interactions in high and low CLIC1 group. **(H)** A barplot reveals the ratio of interaction strength between high and low CLIC1 groups for each signaling pathway. **(I)** Pathway exploration between high and low CLIC1 group through GSVA analysis.

The single-cell dataset GSE138794 comprised of nine samples, which contains 19,315 cells. The number of sequence genes, sequencing depth, and proportion of mitochondrial content were visualized in Supplementary Figure S5A. The sequencing depth and gene numbers presented a strong positive correlation (r = 0.92), while showed a weak correlation with mitochondrial content (Supplementary Figure S5B). Then, 2000 highly variable genes were selected from standardized expression matrix and top 10 genes were labeled (Supplementary Figure S5C). Based upon scRNA profiles, single cells from nine glioma specimens were clustered into 32 cell clusters (Supplementary Figure S5D). In line with known markers of cell types, six cell populations were identified, composed of astrocytes, endothelial cells, glial cell, monocytes (Mono)/macrophages (Macro) and oligodendrocytes (Figure 9A). CLIC1 was found to be expressed ubiquitously in distinct cell types, such as macrophages, astrocytes (Figure 9B). Moreover, we categorized cells into high and low CLIC1 group based on the median expression level of CLIC1. In comparison to the high CLIC1 group, the low expression group has a greater quantity of cells (Figure 9C). The cells in high expression group were mostly concentrated on the macrophages and endothelial cells (Figure 9D). The cell - cell communication indicated that astrocytes and oligodendrocytes showed a strong interaction with other cell types (Figures 9E, F). In addition, the total number of interactions and interaction strength of the inferred cell-cell communication networks were compared between high and low CLIC1 group and revealed that high group showed a higher cell-cell interaction strength (Figure 9G). Then, we identified context-specific signaling pathways by comparing the interaction strength between high and low CLIC1 groups. Specifically, signaling pathways like MHC−II and NOTCH were found to be active in the high CLIC1 group (Figure 9H). Pathway enrichment analysis revealed that IL6_JAK_STAT pathway, TNFA signaling pathway, inflammatory response and KRAS signaling pathway were significantly enriched in high CLIC1 group, revealed that the oncogene role in golima (Figure 9I). To gain a deeper understanding of the differences between cell statuses, we monitored the movement trajectories of different cells. The findings indicated that all cells categorized into one root and three states, names 1, 2 and 3 (Supplementary Figures S6A–C). Moreover, we observed that CLIC1 was highly expressed in beginning of the trajectory (Supplementary Figure S6D). We subsequently using the cytotrace software to infer stemness (less differentiation) of the six cell types. As showed in Supplementary Figure S6E, astrocytes and oligodendrocytes have relative high cytotrace score, indicated that higher stemness of them.

## Discussion

The present work demonstrated that elevated CLIC1 was linked with worse survival outcomes of gliomas. IDH-mutant gliomas usually exhibit low histologic grade with desirable prognostic outcomes and median survival of exceeding 12 years (Nicholson and Fine, 2021). Nevertheless, they usually transition to higher grade and clinical behaviors later in the natural history of this malignancy. Oppositely, IDH–wild-type gliomas often present as GBM (Ostrom et al., 2023). MGMT methylation status represents another molecular feature of gliomas. Herein, CLIC1 expression was elevated in more advanced tumors and IDH–wild-type and MGMT unmethylation status. Thus, CLIC1 was a possible prognostic factor of gliomas.

CLIC1, a member of the CLIC protein family, has emerged as a pivotal player in cancer progression across various malignancies (Ozaki et al., 2022). It exists in both soluble and membrane forms, and its multifaceted involvement in cancer biology has garnered significant attention, particularly in the context of diagnosis and therapeutic targeting. In a multitude of cancer types, CLIC1 has exhibited its potential as a diagnostic indicator and a therapeutic target. It influences fundamental cellular processes, including cell viability and mitochondrial function modulation. In ovarian cancer, CLIC1 has been identified as a promising biomarker (Ye et al., 2015; Singha et al., 2018) with prognostic value (Yu et al., 2018), impacting patient survival and possibly serving in conjunction with CLIC4. Additionally, CLIC1 may have implications in lymphoblastic leukemia (Dehghan-Nayeri et al., 2017). Breast cancer shows heightened CLIC1 expression, correlating with tumor characteristics such as size, TNM classification, pathological grade, lymph node metastasis, and Ki67, while lower expression levels associate with extended overall survival and progression-free survival. This indicates CLIC1’s potential as a diagnostic marker for breast cancer (Xia et al., 2022). In esophageal squamous cell carcinoma (ESCC), CLIC1’s elevated expression aligns with clinical TNM classifications, and its inhibition may promote the mTOR signaling pathway, impacting cell proliferation (Geng et al.). High CLIC1 expression in lung adenocarcinoma has been linked to reduced overall survival, establishing it as an independent prognostic factor (Yasuda et al., 2022). In gastric cancer, the absence of CLIC1 impedes invasion and migration, likely by increasing the expression of AMOT-p130 (Qiu et al., 2021). Hepatocellular carcinoma (HCC) exhibits upregulated CLIC1 expression, associated with tumor invasiveness, metastasis, and poor prognosis (Peng et al., 2021). CLIC1 plays a role in creating a microdomain that facilitates integrin-mediated adhesions and cytoskeletal extension, further contributing to HCC progression.

GBM presents high CLIC1 expression (Wang et al., 2012; Setti et al.), and its suppression reduces proliferation and self-renewal capabilities in glioma stem cells (Setti et al.). GBM aggressiveness correlates with CLIC1-mediated channel activity, suggesting a potential membrane-associated role for CLIC1 in tumor settings. CLIC1’s ability to modulate reactive oxygen species and pH fluctuations influences GBM stem cell motility and proliferation, making it a promising therapeutic target (Peretti et al., 2018). Notably, CLIC1 alterations have been observed in solid tumors and vascular malformations, particularly in GBM and bladder cancers (Bia et al., 2016). Moreover, CLIC1 expression has been linked to the drug-resistant protein MRP1, emphasizing its potential relevance in drug resistance mechanisms (Wu and Wang, 2017). In summary, CLIC1 plays a pivotal role in various cancer types, affecting proliferation, migration, invasion, and metastasis. Targeting CLIC1 holds promise for malignant tumor treatment, although comprehensive mechanistic understanding and targeted therapies necessitate further research. CLIC1’s significance in cancer and glioma progression, as well as its impact on patient survival, present exciting avenues for future cancer research.

Consistent with prior studies (Barbieri et al., 2022; Peretti et al., 2018; Setti et al.), CLIC1 was connected to physiological processes, tumorigenic and immunity-relevant signaling pathways. More importantly, suppressing CLIC1 notably induced glioma cell apoptosis and alleviated cell motility, proving that CLIC1 as a treatment vulnerability of glioma. Glioma progression is shaped by genetic evolution and microenvironment crosstalk (Varn et al., 2022). Detailed characterization of genomic alterations has not determined subtype-specific vulnerabilities in glioma patients (Feng et al., 2023). Heterogeneous genomic alterations were found in low and high CLIC1 expression tumors. Epigenetic remodeling is a molecular hallmark of glioma, which has been regarded as a pivotal mediator of glioma pathogenesis (McClellan et al., 2023). CLIC1 overexpression was potentially affected by altered DNA methylation.

Malignant gliomas remain very difficult to cure because full surgical excision is not biologically feasible owing to the aggressive nature and the proximity of the tumor to functionally sensitive regions (Siegelin et al., 2021). In addition, adjuvant therapy faces frequent treatment resistance because the central nervous system is a protective environment and tumor cells exhibit large intratumor genetic and epigenetic variations. Consequently, novel treatments are urgently required, but the development processes of novel drugs that eventually achieve clinical applications are time-consuming and expensive. High CLIC1 expression was detected to associate with improved sensitivity to chemotherapy and targeted agents (camptothecin, cisplatin, doxorubicin, erlotinib, paclitaxel and rapamycin) as well as small molecular compounds clofarabine, tanespimycin, methotrexate, everolimus, TAK-733, trametinib and AZD8330.

ICB has revolutionized modern cancer treatment, arousing great interest in the field of neuro-oncology. ICB can unleash or redirect the function of T lymphocytes, carrying out cell-mediated immune response that directly kills tumor cells and enhances the anticancer abilities of other immune cell types. For complete anticancer immunity, T cells must have both effector function and the capacity to infiltrate the microenvironmental niche. Despite the well establishment of predictive biomarkers of response to ICB in several cancer types, they are limited to immunogenic cancer types, and malignant gliomas are largely refractory to ICB (Liu et al., 2023). Possible causes contain intrinsic characteristics of glioma cells, e.g., extensive genomic heterogeneity and poor mutation burden, extrinsic characteristics of immunosuppressive microenvironment and microvascular niches (Wu et al., 2023). CLIC1 overexpression was linked with most immune cell populations (notably T cells) and elevated expression of immune-modulators, indicative of a positive interaction of CLIC1 with hot tumors. Based upon high expression of immune checkpoints and similar transcriptome profiling to patients who responded to anti-PD-1, increased expression of CLIC1 indicated well response to ICB. Nevertheless, tumors with CLIC1 upregulation presented elevated TIDE score, indicating that they were resistant to ICB.

Single-cell transcriptomics shows the capacity to resolve whole transcriptomes of single cells with substantial throughput, which has revolutionized research of gene expression (Stuart et al., 2019). CLIC1 was expressed ubiquitously in astrocytes, endothelial cells, malignant cells, Mono/Macro and oligodendrocytes. CLIC1 is required for beta-amyloid-induced production of neurotoxic reactive oxygen species by microglia (Milton et al., 2008). Intracellular CLIC1 modulates macrophage function via mediating phagosomal acidification (Jiang et al., 2012). CLIC1 mediates endothelial S1P receptor to promote Rac1 and RhoA activity and functions (Mao et al., 2021). Thus, CLIC1 not only facilitates glioma tumor growth but also affects the tumor microenvironment.

Altogether, CLIC1 overexpression served as a prognostic factor of glioma patients and mediated malignant progression. It was also linked with genomic alterations, anticancer immunity and immune response as well as drug sensitivity. Our findings revealed that CLIC1 possessed the potential as a treatment vulnerability and actionable target in gliomas.

## Data Availability

The original contributions presented in the study are included in the article/Supplementary Material, further inquiries can be directed to the corresponding author.

## Supplementary material

SUPPLEMENTARY FIGURE S1 Combination of TCGA-GBM and TCGA-LGG samples and correction of batch effects. **(A, B)** PCA plots of TCGA-GBM and TCGA-LGG samples before and after correcting batch effects.

SUPPLEMENTARY FIGURE S2 Verification of OS difference and ROCs in the **(A)** CGGA_325, **(B)** CGGA_693, **(C)** GSE16011 and **(D)** GSE43378 cohorts.

SUPPLEMENTARY FIGURE S3 CLIC1 independently estimates glioma patients’ prognosis. **(A, B)** Uni- and multivariate cox regression results on CLIC1, age, gender, grade, IDH1 and MGMT status with patient survival in TCGA dataset. **(C–F)** Verification of prognostic significance of CLIC1 and these clinicopathological parameters in the **(C, D)** CGGA_325 and **(E, F)** CGGA_693 datasets. Cox univariate regression analysis and multivariate Cox regression analysis were implemented to define the independent prognostic factor for OS.

SUPPLEMENTARY FIGURE S4 CLIC1 up-regulation is linked with hot tumors. **(A)** Different infiltration of immune cells in low and high CLIC1 expression tumors. **(B)** Interactions of CLIC1 with immune cell infiltration. **(C–F)** Expression patterns of **(C)** chemokine, **(D)** receptor, **(E)** immuno-stimulator and **(F)** MHC molecules in two groups. Blue to red denotes lowly to highly expressed molecules. The Student’s t-test was used to compare the statistical difference between two groups. ****p*-value < 0.001.

SUPPLEMENTARY FIGURE S5 Quality control and normalization of GSE138794 dataset. **(A)** Quality assurance of scRNA-Seq data derived from 9 golima samples. **(B)** Correlation between the detected gene numbers, mitochondrial content, and sequencing depth **(C)** Characterization of top 2,000 highly variable genes in scRNA-seq data. **(D)** TSNE and UMAP for single cell clustering analysis based upon scRNA data from the GSE138794.

SUPPLEMENTARY FIGURE S6 Differentiation trajectory analysis of scRNA-Seq data from the GSE138794. **(A)** Visualization of the trophoblast states throughout the trajectory analysis. **(B)** The biaxial scatter plot illustrates the development of trophoblastic cells, with darker shades denoting the earlier stages. **(C)** A sequence of cell clusters is plotted along pseudo-time, with each point representing a cell and each color representing a state. The cell sequence can be determined by examining the expression of the most dispersed genes in the cell cluster. **(D)** The expression of CLIC1 is plotted along pseudo-time, with each point representing a cell. **(D)** Trajectories of differentiation and distribution of six cell type were derived by CytoTRACE.

SUPPLEMENTARY TABLE S1 Clinical traits of glioma patients in TCGA, CGGA_325, CGGA_693, GSE16011 and GSE43378.

## Abbreviations

AUC, area under the curve; CGGA, the Chinese Glioma Genome Atlas; CLIC1, Chloride intracellular channel 1; DSS, disease-specific survival; ESCC, esophageal squamous cell carcinoma; GBM, glioblastoma multiforme; GSEA, gene set enrichment analysis; HCC, hepatocellular carcinoma; HGG, high-grade glioma; IC50, half-maximal inhibitory concentration; ICB, immune-checkpoint blockade; LGG, low-grade glioma; MGMT, O6-methylguanine DNA methyltransferase; OS, overall survival; PFS, progression-free survival; ROCs, receiver operating characteristic curves; RT-qPCR, Real-time quantitative PCR; ssGSEA, single-sample GSEA; TCGA, the Cancer Genome Atlas; TIDE, Tumor Immune Dysfunction and Exclusion; TMB, tumor mutational burden; WHO, World Health Organization.
