# Supplementary figures and images for: Multi-omics analysis reveals CLIC1 as a therapeutic vulnerability of gliomas
Source: Front Pharmacol. 2023 Nov 8;14:1279370. doi: 10.3389/fphar.2023.1279370 (PMC10663228; doi:10.3389/fphar.2023.1279370)

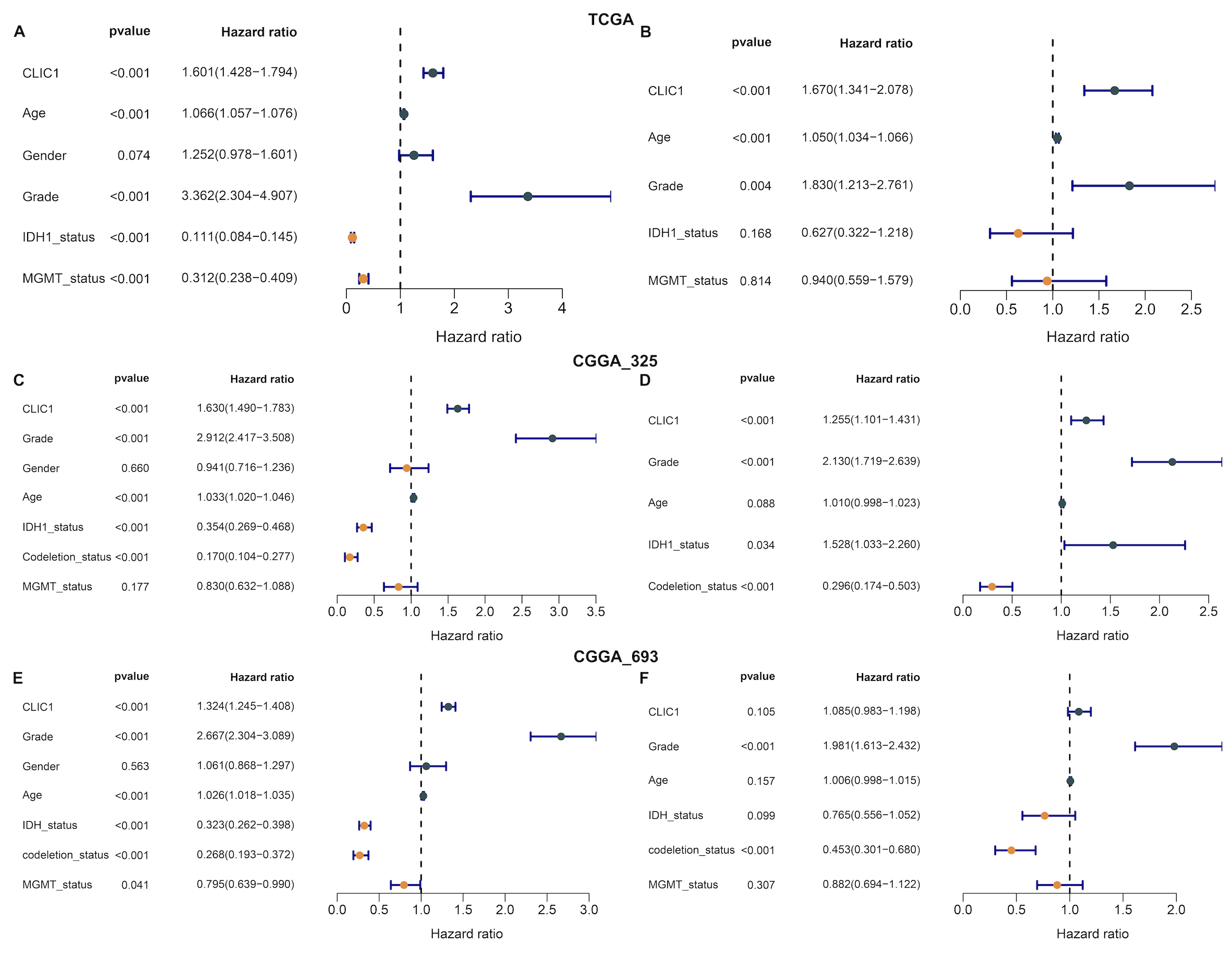

Supplement: Supplementary file 1 [file Image3.TIF]

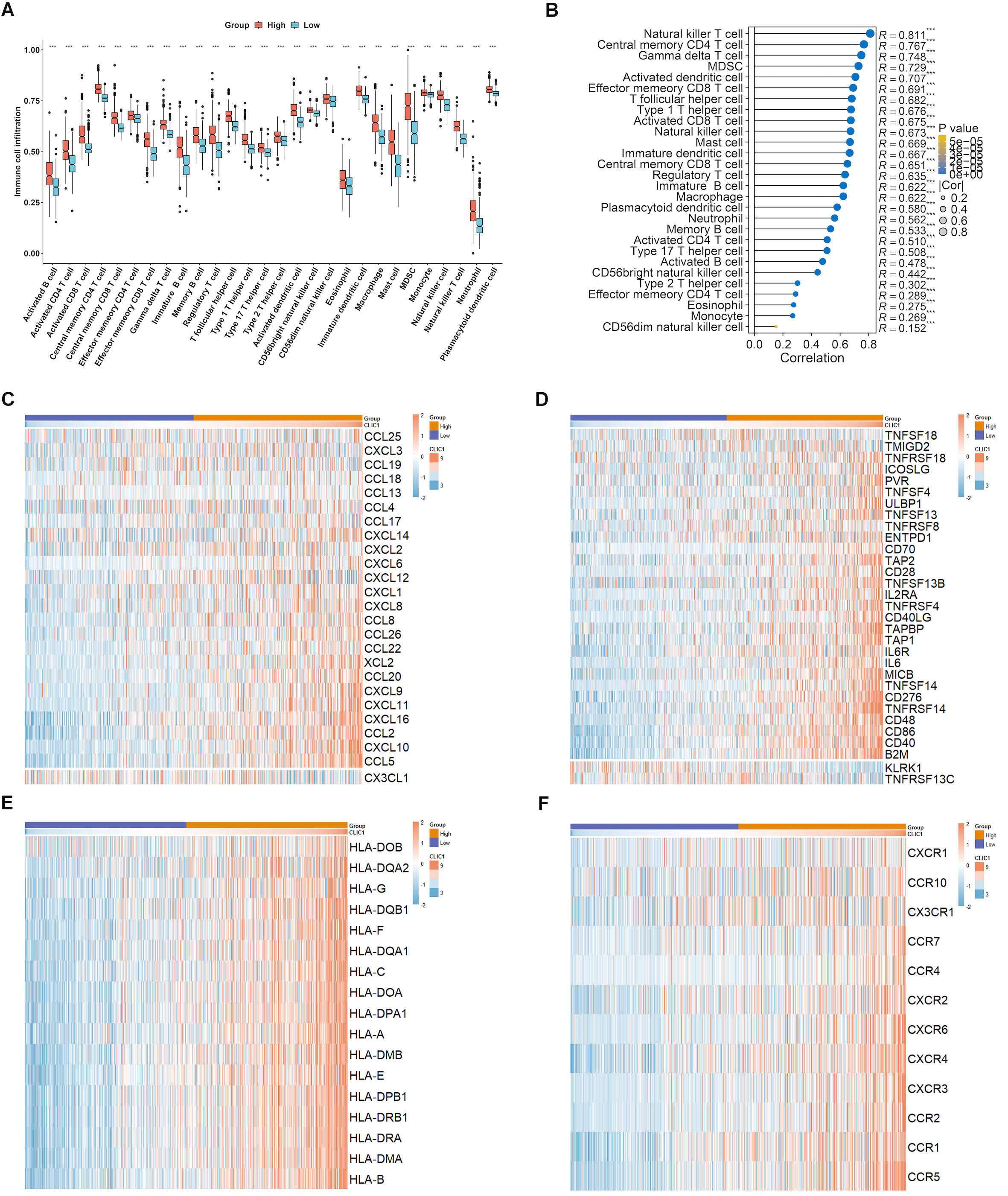

Supplement: Supplementary file 2 [file Image4.JPEG]

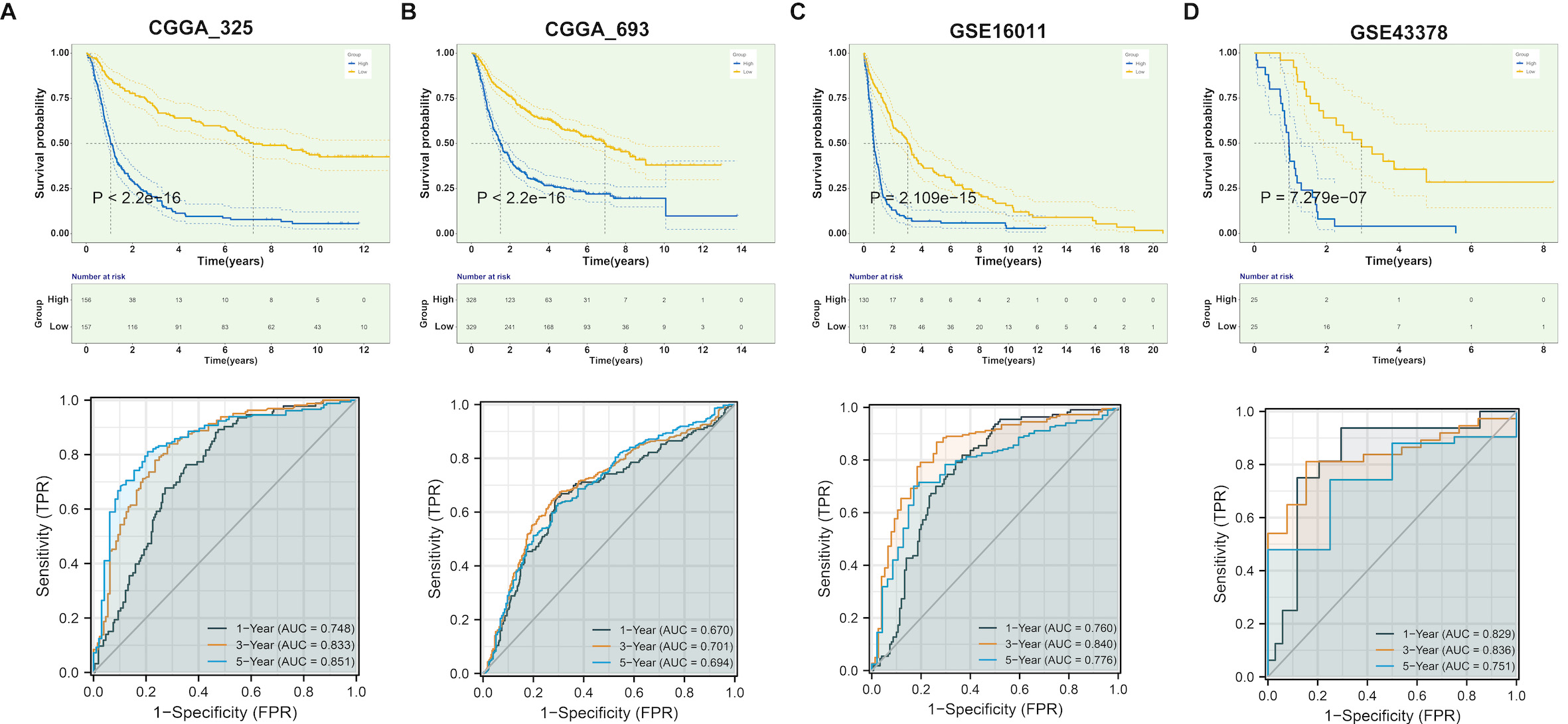

Supplement: Supplementary file 3 [file Image2.JPEG]

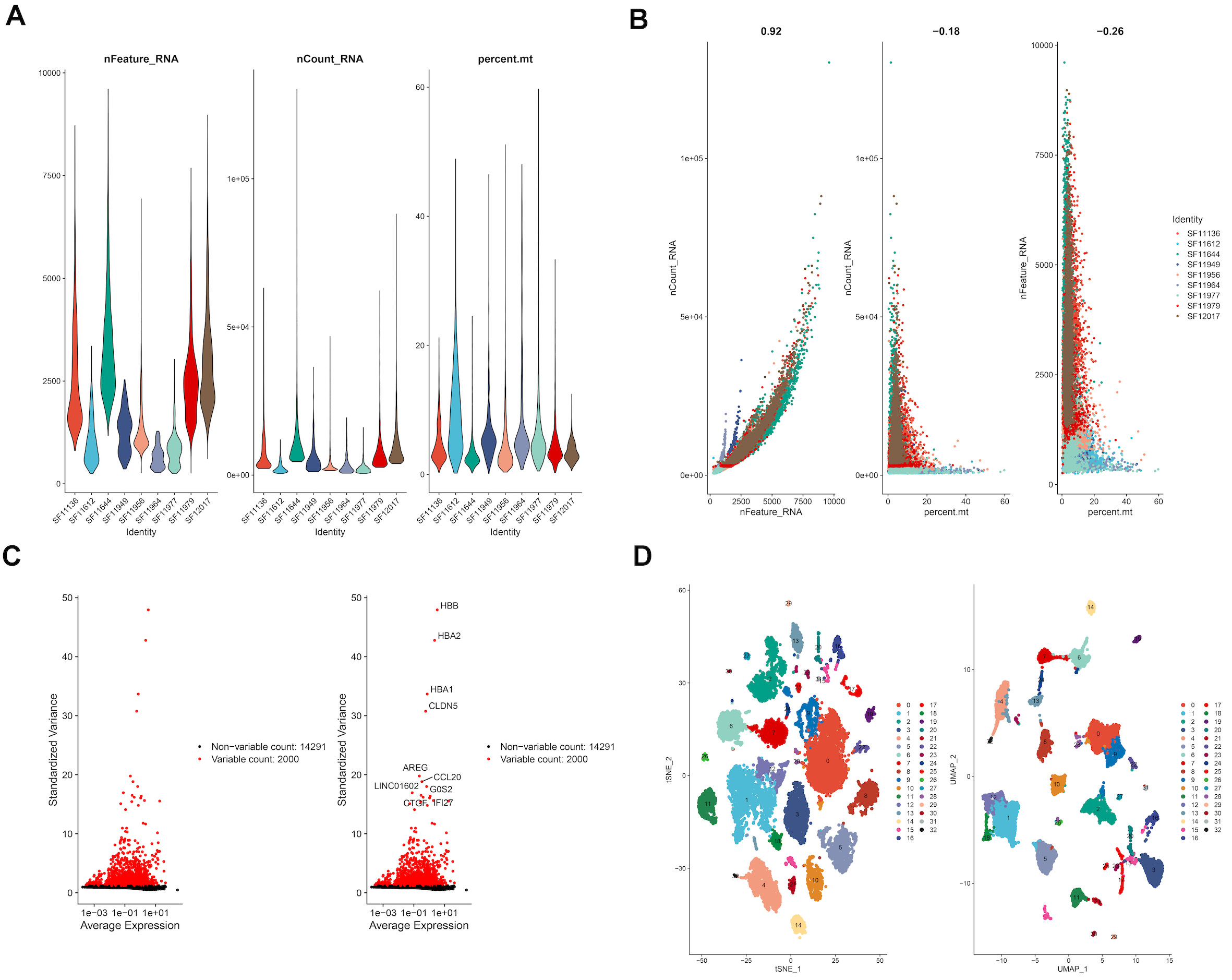

Supplement: Supplementary file 4 [file Image5.JPEG]

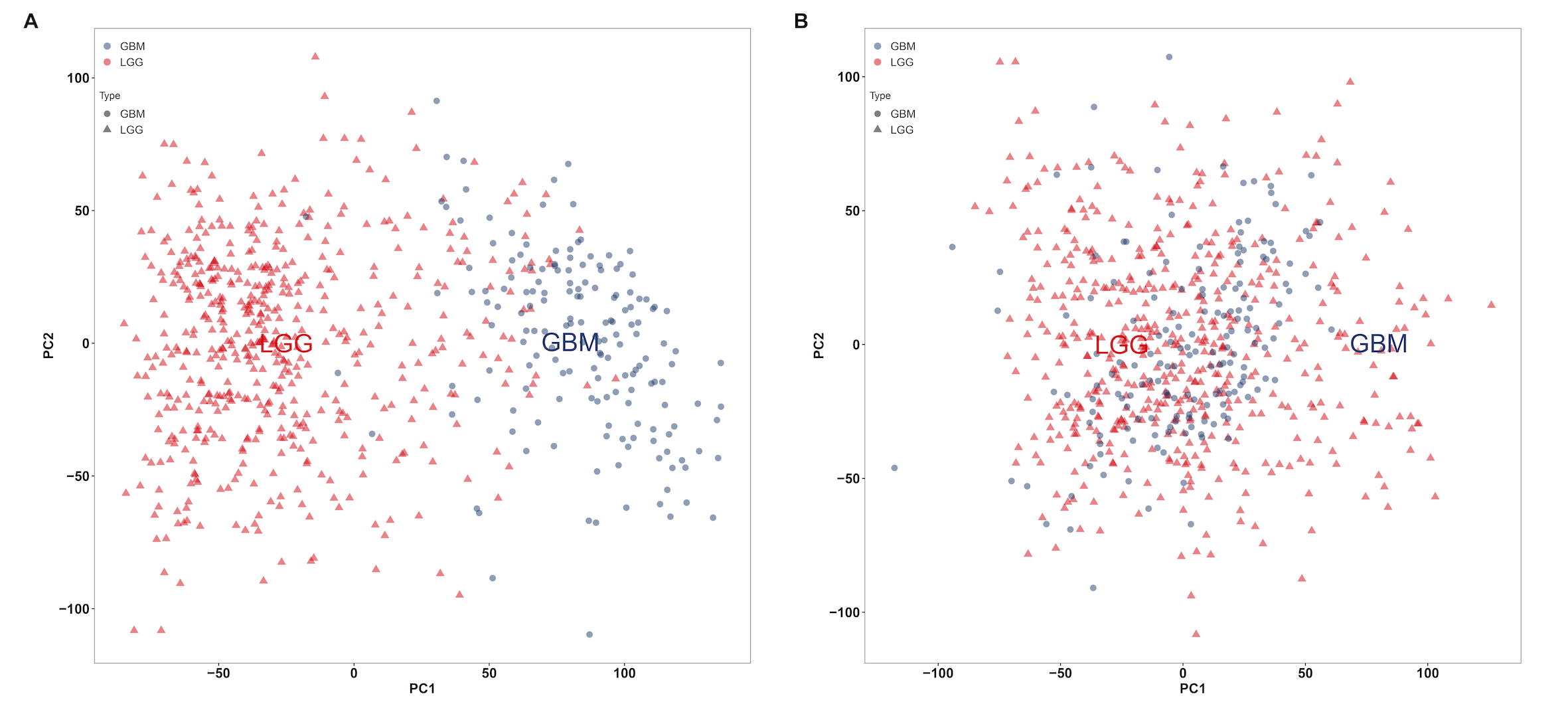

Supplement: Supplementary file 5 [file Image1.TIF]

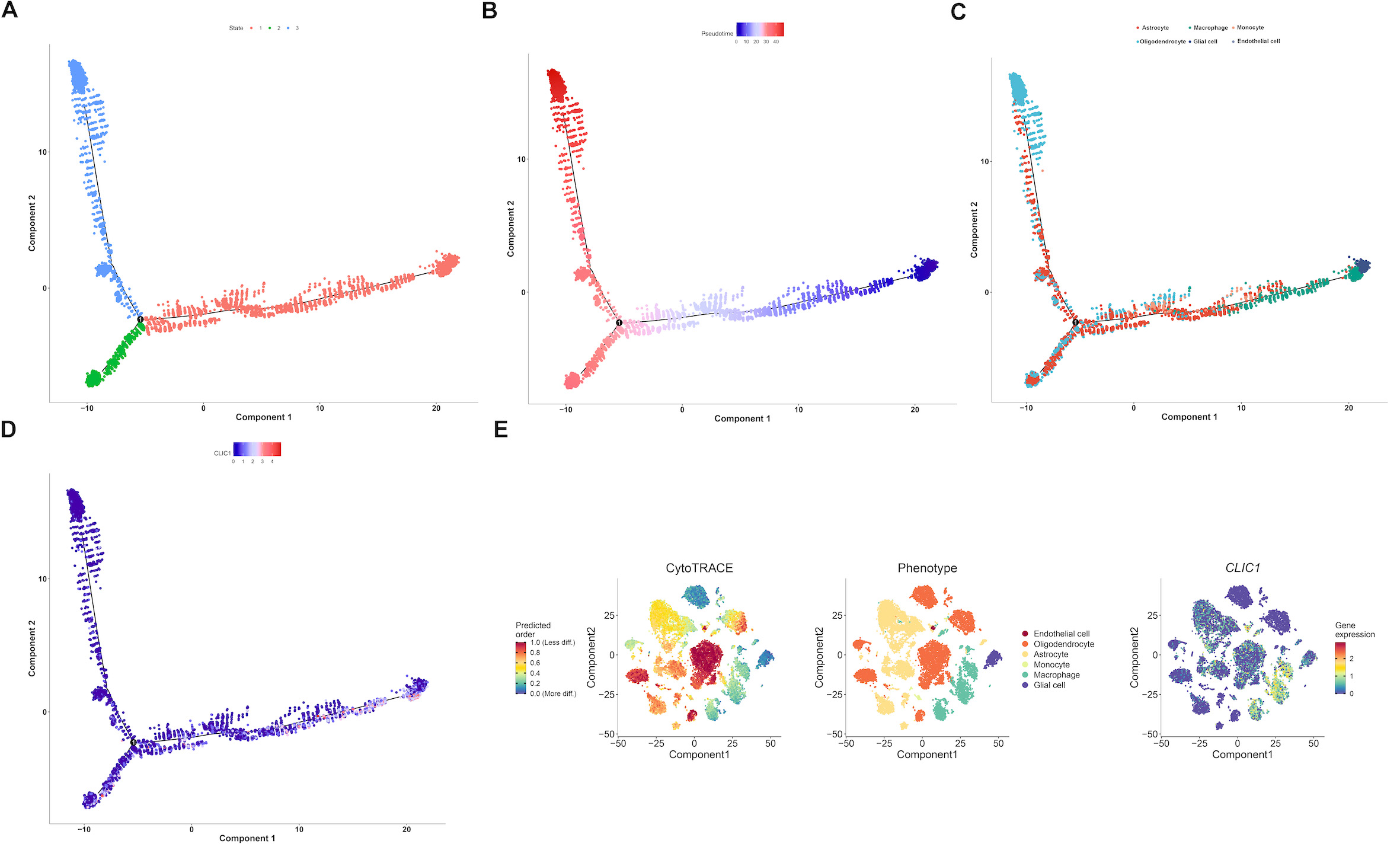

Supplement: Supplementary file 7 [file Image6.JPEG]
